# Type IV Paraesophageal Hernia With Posterior and Inferior Rotation of the Stomach: A Case Report

**DOI:** 10.7759/cureus.64417

**Published:** 2024-07-12

**Authors:** Nicolas D Benelli, Braxton Altom, Jacqueline Gomez-Garzon, Juan Matamoros, Steven Perez-Bello, Lissette Castellanos, Carlos Bello

**Affiliations:** 1 Internal Medicine, St. George's University School of Medicine, St. George's, GRD; 2 Emergency Medicine, St. George's University School of Medicine, St. George's, GRD; 3 Internal Medicine, Keralty Hospital Miami, Miami, USA; 4 Biology, Florida International University, Miami, USA; 5 Internal Medicine, Florida International University, Miami, USA; 6 General Surgery, Keralty Hospital Miami, Miami, USA

**Keywords:** gastric displacement, hernia, gastric herniation, da vinci robotic system, paraesophageal hernia

## Abstract

This case report describes a patient with a large type IV hiatal hernia (HH), notable for exhibiting minimal symptoms, unlike typical cases of similar severity. The patient experienced only mild discomfort despite significant anatomical displacement, without severe symptoms often seen with such hernias. Diagnostic tests confirmed the herniated stomach, but the lack of severe symptoms like dysphagia defies usual expectations. This case highlights the variability in symptoms and clinical presentations of HH, stressing the need for tailored assessment and management for each patient.

## Introduction

The diaphragm, a vital muscle for respiration, features an opening known as the esophageal hiatus, allowing the esophagus to pass from the thorax to the abdomen. At this junction, the lower esophageal sphincter acts as a barrier against gastric reflux into the esophagus to prevent acidic stress to the esophageal mucosa. A hiatal hernia (HH) occurs when part of the stomach protrudes through the diaphragmatic esophageal hiatus and into the thoracic cavity [1]. This herniation may compromise the normal anatomy and function of the upper gastrointestinal tract.

An HH may be classified based on the extent of herniation and the position of the gastroesophageal junction. The degree of herniation is used to classify an HH from types I-IV. The most prevalent form is the type 1 sliding HH [1]. Type IV HH, the rarest yet most complicated presentation, results from herniation of additional abdominal structures including the entire stomach, intestine, and spleen [2].

The patient presentation will depend on the severity of the herniation. Type I HH usually is asymptomatic and discovered incidentally on imaging. Types II-IV HH may present with epigastric pain, postprandial dyspnea, early satiety, vomiting, and nausea. Symptoms of gastroesophageal reflux disease (GERD) may also be present, and this includes and is not limited to dyspepsia, odynophagia, halitosis, and retrosternal burning pain [3].

Management of HH is dependent on symptoms and anatomical presentation. Lifestyle adjustments such as dietary modifications and elevation of the head while sleeping are recommended to mitigate symptoms associated with GERD. Pharmacological interventions may include proton pump inhibitors (PPIs) and histamine receptor antagonists. These medications are typically reserved for patients who have symptoms refractory to conservative measures [4]. Surgical intervention is warranted for a severely symptomatic or complicated HH. These measures are particularly utilized in the treatment of paraesophageal hernias. Surgical methods include laparoscopic or open Nissen fundoplication, which aims to reinforce the lower esophageal sphincter and prevent reflux by wrapping the fundus of the stomach around the esophagus. Surgical treatment of a paraesophageal HH may include reducing the hernia sac and repairing the diaphragmatic defect to avert complications such as volvulus or strangulation [5].

This case has been investigated to emphasize the ability of an HH, even with prominent displacement, to go unnoticed due to a lack of major symptom presentation. Clinicians may approach patients who present with mild gastrointestinal symptoms in a conservative manner when really there could be a major anatomical anomaly causing the symptoms with the potential to cause life-threatening complications.

## Case presentation

**Figure 1:**
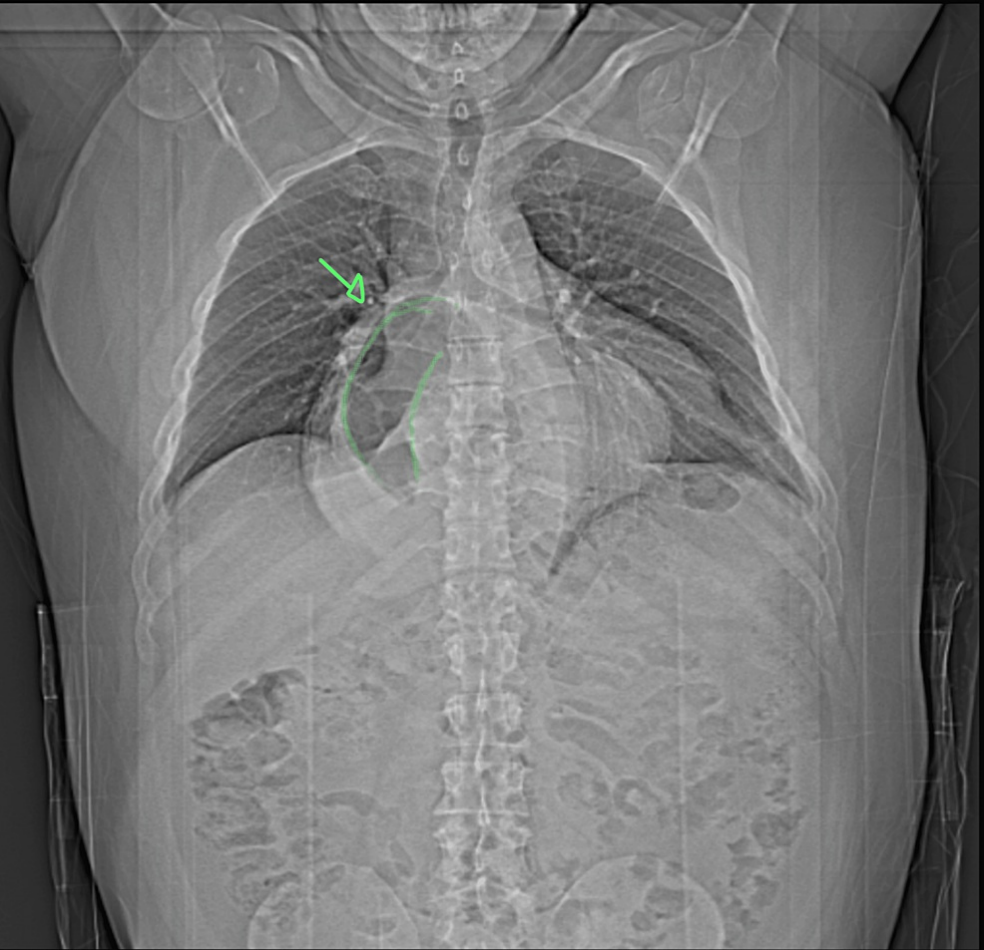
Preoperative anterior-posterior chest X-ray The green arrow and green outline indicate the stomach.

**Figure 2:**
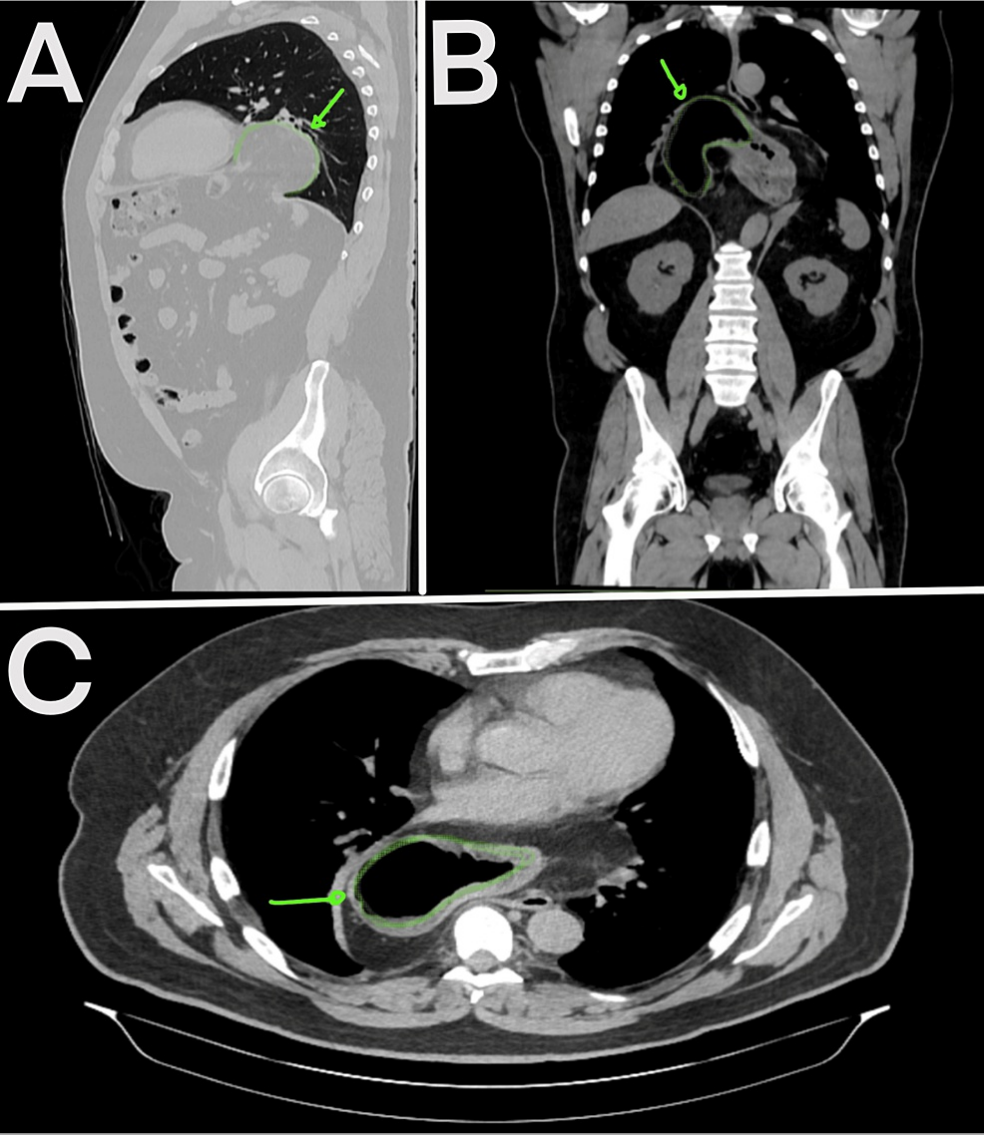
Preoperative computed tomography A: Sagittal view; B: coronal view; C: axial view; the green arrow and green outline indicate the stomach.

Prior to the writing of this report, verbal consent was obtained from the patient. The patient is a middle-aged male who presented with a six-month history of bloating that was especially pronounced about half an hour after consuming meals. Apart from intermittent heartburn being treated with omeprazole, the patient had no other pertinent medical conditions. The patient often found himself waking up in the middle of the night several times to induce vomiting which relieved the bloating. The vomiting was non-billous and non-bloody. He has not had severe dysphagia, abnormal weight loss, or hemoptysis. When the symptoms began, the patient initially believed they were side effects from his semaglutide medication being used for weight loss. However, the progression of bloating prompted the patient to seek medical evaluation. A decision was made for the patient to undergo imaging to evaluate for any underlying pathologies. An endoscopy revealed a type IV HH with posterior and inferior rotation of the stomach into the thoracic cavity. These findings were further supported by X-ray imaging and computed tomography (CT) (Figures 1-2).

**Figure 3:**
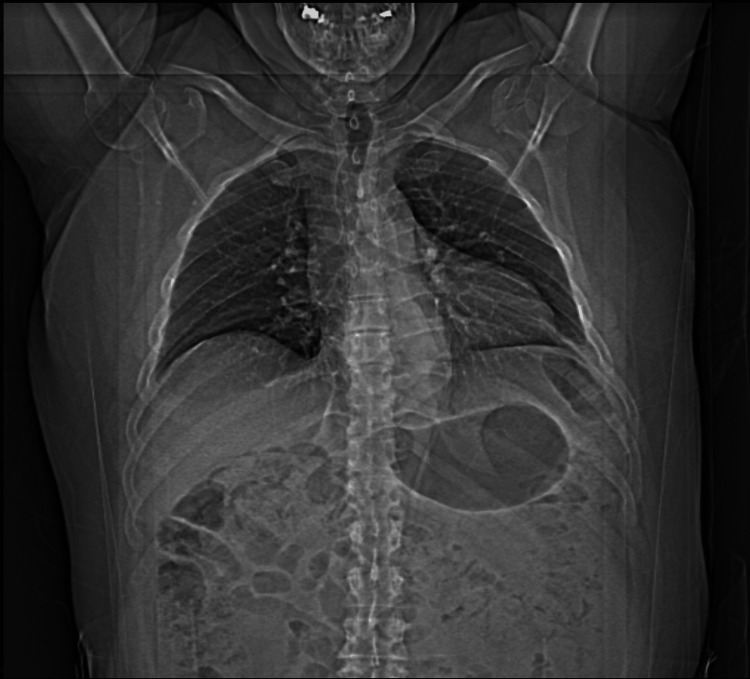
Postoperative chest X-ray after hernia repair

**Figure 4:**
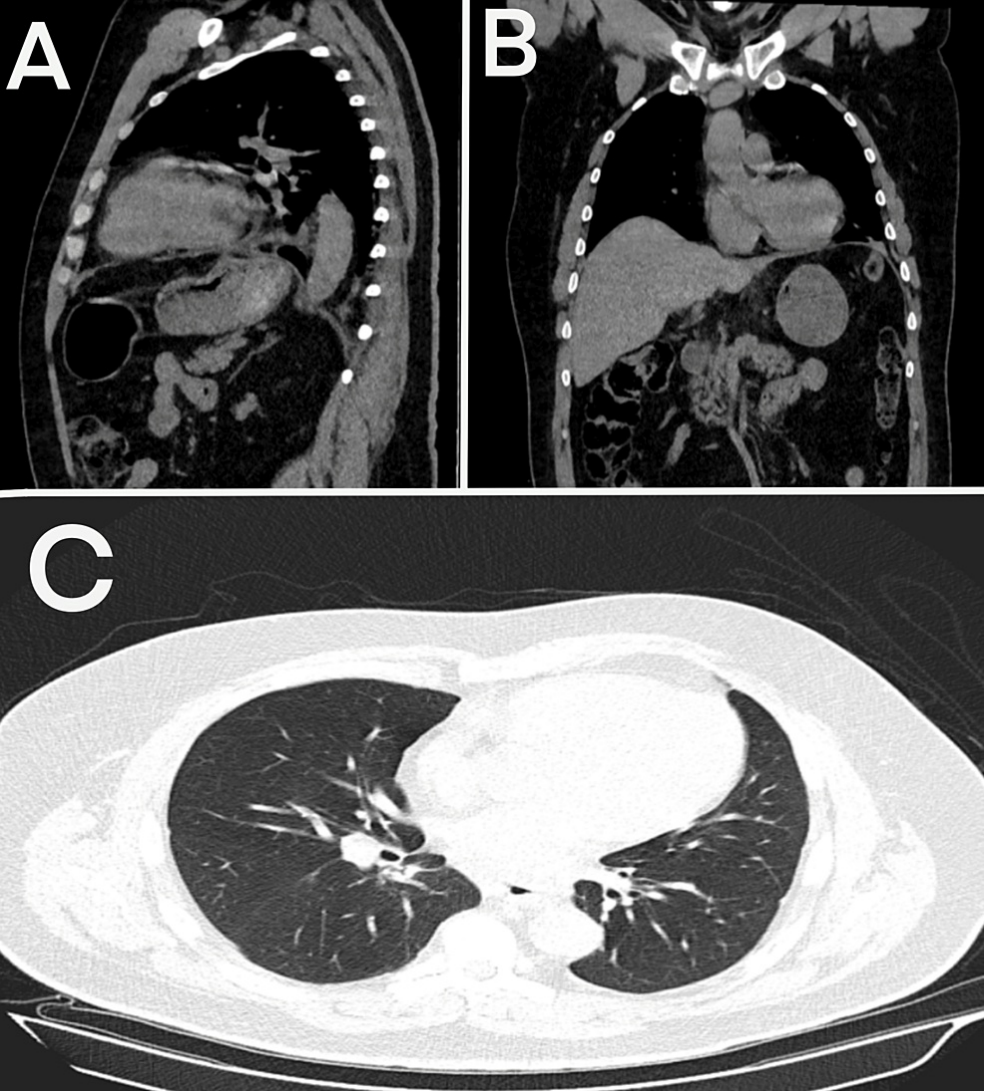
Postoperative computed tomography after hernia repair A: Sagittal view; B: coronal view; C: axial view

The results from imaging warranted surgical intervention to treat the hernia and prevent any further complications. The surgery was performed robotically with the Da Vinci XI (Intuitive Surgical, Inc., Sunnyvale, USA). After the reduction of the stomach in its entirety, the hernia sac was dissected and resected. Dissection of the mediastinum was done in a way to allow for 2-3 centimeters of intra-abdominal esophagus. A combination of barbed Prolene (Ethicon, Inc., Somerville, USA) and polyester sutures was used to repair the hiatus, which was later reinforced with an absorbable mesh. A Toupet fundoplication was then done, and the integrity of the operation was confirmed via endoscopy (https://www.youtube.com/watch?v=mwuP3sAxtwc). After the operation, the patient underwent repeated imaging to assess the correction of the hernia as seen in Figures 3-4.

## Discussion

A type IV HH with such a significant degree of gastric displacement is unusual and would typically present with more prominent and severe symptoms than those observed in this patient. Symptoms commonly associated with a large type IV HH can include severe chest pain, difficulty swallowing, chronic acid reflux, shortness of breath due to the compression of lung structures, and even palpitations if the heart is affected [6]. The relatively mild symptoms in this patient suggest a gradual adaptation of the body to the hernia. Limitations of this study may include attempting to generalize the findings of this patient to others who have an HH. It would not generally be recommended for patients to undergo extensive workups if the symptom presentation is minimal.

One critical factor contributing to the delayed diagnosis and mild presentation is the absence of imaging studies in the patient's medical history. Without previous imaging, it is challenging to determine the duration of the hernia and its progression over time. The insidious nature of type IV HH means it can grow significantly without causing acute symptoms until it reaches a critical size or triggers a specific complication.

The recent prescription of semaglutide, a medication commonly used to manage type 2 diabetes and obesity by inducing weight loss and slowing gastric emptying, likely exacerbated the patient's symptoms. Semaglutide’s effects on gastric motility might have increased intragastric pressure, pushing more of the stomach into the thoracic cavity and exacerbating the hernia's symptoms.

Type IV HH is particularly concerning due to its predisposition to life-threatening complications. Gastric volvulus, a condition where the stomach twists upon itself, is one such risk. This twisting can lead to strangulation of the stomach tissue, cutting off blood supply, and resulting in gastric gangrene or perforation. Both conditions are surgical emergencies with high morbidity and mortality rates [3].

In addition to gastric complications, cardiothoracic complications are also significant concerns. There was a notable case report published in 2020 describing a patient with type IV HH who experienced hemodynamic instability. In this instance, the hernia was so extensive that it caused compression of the left atrium, leading to a compromised cardiac function [6]. This scenario underscores the potential severity of type IV HH and the necessity for prompt and effective management.

Surgical intervention is often required for managing type IV HH, and there are several approaches to consider. The primary surgical techniques are open surgery, laparoscopic surgery, and robotic-assisted surgery. Each method has its indications, benefits, and drawbacks. Open surgery, once the standard for HH repair, is now generally considered outdated as an initial approach due to its higher morbidity and mortality rates compared to minimally invasive techniques [7]. However, it may still be used in complex cases or when minimally invasive surgery is not feasible.

Laparoscopic surgery has been the preferred approach for the past few decades. It involves making several small incisions through which a camera and surgical instruments are inserted. This technique has the advantage of being less invasive, with a shorter recovery time and reduced postoperative pain compared to open surgery [8]. However, it requires a high degree of skill and experience from the surgeon, particularly for large or complicated hernias like type IV HH.

Robotic-assisted surgery is a relatively new technique that has shown promise in the repair of paraesophageal hernias, including type IV HH. This approach uses robotic arms controlled by the surgeon, offering greater precision, flexibility, and control than traditional laparoscopic surgery. Studies have shown that robotic surgery can lead to improved postoperative recovery and fewer complications. For example, patients undergoing robotic surgery for HH repair tend to have shorter hospital stays and less postoperative pain. Despite these advantages, robotic surgery also comes with higher costs and longer operative times, which must be weighed against its benefits [9].

When deciding between laparoscopic and robotic surgery, several factors need to be considered. The patient’s age, overall health, past medical history, and presence of co-morbidities play crucial roles in determining the most appropriate surgical approach. For instance, older patients or those with significant co-morbidities may benefit more from the minimally invasive nature of robotic surgery, despite the higher cost. Additionally, the surgeon's expertise and familiarity with each technique are important considerations, as the success of the surgery heavily depends on the skill and experience of the surgical team [8].

In conclusion, the management of type IV HH requires a thorough understanding of the potential complications and a careful consideration of the available surgical options. While laparoscopic surgery remains the standard of care, robotic-assisted surgery is emerging as a valuable alternative, particularly for complex cases. Both approaches offer safe and effective outcomes, but the choice between them should be individualized based on the patient's specific circumstances and the surgeon’s expertise. Ongoing advancements in surgical technology and techniques continue to improve the outcomes for patients with type IV HH, offering hope for less invasive and more effective treatments in the future.

## Conclusions

In conclusion, type IV HH represents a complex condition with potentially life-threatening complications, necessitating prompt diagnosis and appropriate management. This case report underlines the importance of clinicians considering an HH in patients who present with only minimal or even no symptoms, as lack of treatment may result in detrimental complications. Surgical correction in this case had to be cautiously planned due to the complex nature of the patient's herniation.
